# Emotion Perception Mediates the Predictive Relationship Between Verbal Ability and Functional Outcome in High-Functioning Adults with Autism Spectrum Disorder

**DOI:** 10.1007/s10803-017-3036-1

**Published:** 2017-02-13

**Authors:** Sadao Otsuka, Shota Uono, Sayaka Yoshimura, Shuo Zhao, Motomi Toichi

**Affiliations:** 1 Faculty of Human Health Sciences, Graduate School of Medicine, Kyoto University, 53 Shogoin Kawahara-cho, Sakyo-ku, Kyoto, 606-8507 Japan; 2 Department of Neurodevelopmental Psychiatry, Habilitation and Rehabilitation, Faculty of Human Health Sciences, Graduate School of Medicine, Kyoto University, 53 Shogoin Kawahara-cho, Sakyo-ku, Kyoto, 606-8507 Japan; 3 The Organization for Promoting Neurodevelopmental Disorder Research (OPNDR), 40 Shogoin Sanno-cho, Sakyo-ku, Kyoto, 606-8392 Japan; 4 International Research Fellow of the Japan Society for the Promotion of Science (JSPS), 5-3-1, Kojimachi, Chiyoda-ku, 102-0083 Tokyo Japan

**Keywords:** Autism spectrum disorder (ASD), Social cognition, Emotion recognition, Adaptive behavior, Social functioning, Predictor

## Abstract

The aim of this study was to identify specific cognitive abilities that predict functional outcome in high-functioning adults with autism spectrum disorder (ASD), and to clarify the contribution of those abilities and their relationships. In total, 41 adults with ASD performed cognitive tasks in a broad range of neuro- and social cognitive domains, and information concerning functional outcomes was obtained. Regression analyses revealed that emotion perception and verbal generativity predicted adaptive functioning directly, and the former mediated between the other two. These findings provide the first evidence of a triadic relationship among neuro- and social cognition and functional outcome in this population. Our results suggest that psychosocial interventions targeting these cognitive abilities could benefit social adaptation in adults with ASD.

## Introduction

Autism spectrum disorder (ASD) is a lifelong neurodevelopmental disorder characterized by deficits in social communication and social interaction, coupled with restricted, repetitive patterns of behavior, interests, or activities (American Psychiatric Association 2013). ASD is a heterogeneous condition in terms of severity and the type of symptoms. Some individuals with subtle or inconspicuous symptoms are not identified until as late as adulthood, as a result of failure to detect them early (Begeer et al. 2013; Lai et al. 2014, 2015; Lehnhardt et al. 2016; National Collaborating Centre for Mental Health 2012). Reflecting the heterogeneity of characteristics in individuals with ASD, there is also considerable variation in outcomes. Nevertheless, their adaptive behavior skills are typically lower than predicted by cognitive capacity, and social functioning in adulthood, including independent living, employment, friendships, and intimate relationships, is generally poor (for a review, see Howlin and Moss 2012). Although the symptoms may be inconspicuous, ASD can affect adaptive and social functioning in individuals with the condition over the course of their life. In terms of prognostic predictions and developing effective interventions for individuals with ASD, it is important to identify predictors of functional outcome, including adaptive and social functioning, both of which correlate closely in individuals with ASD (Farley et al. 2009).

Previous studies have demonstrated that general cognitive ability during childhood (e.g., intelligence quotient: IQ; developmental quotient: DQ), is the strongest predictor of both adaptive and social functioning in adults with ASD (for a review, see Magiati et al. 2014). Limited cognitive ability is likely to worsen functional outcome. However, as Howlin et al. (2004) showed, social functioning in over half of adults with both childhood verbal and performance IQs ≥70 did not differ from that in individuals with an intellectual disability, indicating that a better outcome in adults with ASD was not necessarily guaranteed by an average or higher IQ in childhood. Adaptive and social functioning in individuals with ASD, but without an intellectual disability, may be more variable and less predictable than previously thought. Although only a few studies have examined the predictive value of specific cognitive abilities, they suggest that atypical characteristics in various domains of neuro- and social cognition may predict functional outcomes in the case of high-functioning children with ASD. A cross-sectional study found that verbal abilities, including verbal learning, vocabulary, and spelling, predicted adaptive functioning in high-functioning children with ASD more strongly than IQ (Liss et al. 2001). Thus, investigations into childhood cognitive predictors of functional outcome are warranted. Additionally, it appears to be beneficial to focus on cognitive abilities in adulthood, considering the later diagnosis of high-functioning individuals, expanded deficits in adaptive functioning in adults (Kanne et al. 2011; Klin et al. 2007; Matthews et al. 2015; Perry et al. 2009), the largely unmet needs of support for adults (Kogan et al. 2008; Shattuck et al. 2012), and limited efforts to develop psychosocial interventions for adults (Bishop-Fitzpatrick et al. 2013; Spain et al. 2015; Spain and Blainey 2015). To identify possible targets for psychosocial interventions, in the present study, we investigated predictive relationships between atypical characteristics in cognition and functional outcome in high-functioning adults with ASD.

First, it is necessary to begin by detecting specific cognitive abilities predicting, or accounting for the variability in, functional outcome. Some studies have reported that specific cognitive abilities relate to adaptive or social functioning in adults. Regarding neurocognition, Berger et al. (2003) found that cognitive flexibility (shifting), evaluated on card sorting tests and other tasks, was related to longitudinal changes in adaptive functioning. For social cognition, Wallace et al. (2011) found that performance in facial emotion recognition (FER) correlated positively with adaptive functioning. Montgomery et al. (2013) reported that mentalizing (theory of mind) evaluated on the “Reading the Mind in the Eyes” test, revised version (Eyes Test; Baron-Cohen et al. 2001a), predicted self-reported social stress. These pioneering studies used only a narrow selection of cognitive domains, making it difficult to compare the predictive powers of the various abilities. To identify specific cognitive abilities as targets for psychosocial interventions, it is essential to examine simultaneously the predictive values of a broad range of domains in both neuro- and social cognition in which atypicality has been found in individuals with ASD.

Second, to determine how various cognitive abilities contribute to functional outcome, it is important to consider the intertwined links between abilities, especially between neuro- and social cognition. Previous studies have reported a link between mentalizing and executive functions in individuals with ASD (Ozonoff et al. 1991; Pellicano 2007). White (2013) suggested that poor performance in executive function tasks may be secondary to mentalizing difficulties. However, Pellicano (2010) found that executive functions longitudinally predicted mentalizing performance in children with ASD, suggesting that executive dysfunction underlies social communication impairment. Consistent with this, it has been suggested that explicit cognitive or verbally mediated processing compensates for inefficiency in FER in individuals with ASD (Harms et al. 2010). Some evidence also suggests that individuals with ASD use local processing, or a feature-based strategy, in FER tasks, in contrast to the global, configural-based processing used by typically developing individuals (Behrmann et al. 2006; Rutherford and McIntosh 2007; Walsh et al. 2014). Furthermore, previous studies have reported that general cognitive or language ability predicts FER performance in children with ASD, but not typically developing children (Dyck et al. 2006; Hobson 1986). These findings suggest the possibility that abilities in social cognition, which predict adaptive functioning and are predicted by neurocognitive abilities, may mediate a predictive relationship between neurocognition and functional outcome in adults with ASD. Additionally, atypical and compensatory relationships between specific abilities in neuro- and social cognition may contribute to social adaptation. To our knowledge, no reported study had examined a triadic relationship among neuro- and social cognition and functional outcome in this population.

The primary purpose of the present study was to identify specific cognitive abilities predicting, or accounting for variance in, functional outcome in adults with ASD and average or higher IQ, focusing on the heterogeneity of characteristics and outcome in this population. In this cross-sectional study, we investigated the predictive relationship among specific abilities in neuro- and social cognition and adaptive and social functioning. Specifically, we examined whether specific abilities in neuro- and social cognition could predict functional outcome or whether social cognition would act as a mediator, to clarify the contribution of those abilities and their relationships. As a variable representing functional outcome, we used adaptive functioning, where substantial variance has been found to be explained by specific cognitive abilities (38–64%; Liss et al. 2001). Before examining cognitive predictors, we saw a relationship between adaptive and social functioning, to validate that adaptive functioning was an indicator of functional outcome. We intended to take preliminary steps towards developing evidence-based interventions that promote adaptive and social functioning in adults with ASD. The cognitive abilities predicting functional outcome could be possible targets for psychosocial interventions in this population. We hypothesized that (1) adaptive functioning would predict social functioning, (2) a combination of specific abilities in neuro- and social cognition could account for variance in adaptive functioning, and (3) social cognition would mediate the relationship between neurocognition and functional outcome.

## Methods

### Participants

The ASD group consisted of 41 adults with no intellectual (full-scale IQ ≥ 70) or language disability (verbal IQ ≥ 70) aged 18–53 years (22 males, 19 females), who had been referred to Kyoto University for consultation or cognitive assessments by affiliated hospitals, public consultation offices, or public organization for employment. The ASD participants were diagnosed with autistic disorder (*n* = 1), Asperger’s disorder (*n* = 21), or pervasive developmental disorder, not otherwise specified (*n* = 19), according to the DSM-IV-TR criteria (American Psychiatric Association 2000), by psychiatrists with expertise in developmental disorders, based on an interview with the participants and information from their parents, professionals who helped them, and a clinical record of childhood, when available.

The symptom severity of the ASD participants was assessed by the psychiatrists who made the diagnosis, using the Childhood Autism Rating Scale-Tokyo version (Kurita et al. 1989), which is the Japanese version of the Childhood Autism Rating Scale (CARS; Schopler et al. 1986) and the Childhood Autism Rating Scale second edition, High functioning version (CARS2-HF; Schopler et al. 2010). The CARS and the CARS2-HF include 15 items that assess autism-related behaviors. Total CARS and CARS2-HF scores are the sum of scores on all items and range from 15.0 to 60.0, with higher scores indicating more severe symptoms. The CARS has been shown to be a useful tool for diagnosing autism in children, adolescents, and adults (Mesibov et al. 1989). The scores of participants in the present study were comparable to those of individuals with high-functioning ASD (mean scores ± SDs were 22.22 ± 3.57 in individuals with Asperger’s syndrome and 23.61 ± 3.42 in individuals with high-functioning autism) reported by Koyama et al. (2007). Although our participants’ mean score on the CARS, 24.7, were less than the clinical cut-off (27.0) for a diagnosis of autistic disorder (see Mesibov et al. 1989), participants’ mean score on the CARS2-HF, 30.0, was higher than the cut-off for ASD (28.0). These data indicated that the symptoms of the ASD participants were severe enough to warrant a diagnosis of ASD. The CARS and CARS2-HF scores of participants are presented in Table 1.

**Table 1:** Demographic, clinical, and cognitive characteristics of participants in each group

	ASD (*n* = 41) Mean (SD)	ASD-ND (*n* = 21) Mean (SD)	CON (*n* = 21) Mean (SD)	ASD versus CON			ASD-ND versus CON		
				Statistic	*p*	ES	Statistic	*p*	ES
Demographics									
Age (years)	27.73 (7.91)	25.24 (5.75)	24.90 (6.32)	*t*(60) = 1.42	0.123	*d* = 0.40	*t*(40) = 0.18	0.677	*d* = 0.06
Gender (% male)	53.7%	66.7%	66.7%	χ^2^	0.418		χ^2^	1.000	
Education (years)	15.24 (1.92)	14.95 (1.96)	15.05 (2.29)	*t*(60) = 0.36	0.249	*d* = 0.09	*t*(40) = −0.15	0.661	*d* = −0.04
Clinical characteristics									
CARS	24.70 (2.89)	23.93 (2.88)							
CARS2-HF	30.00 (3.86)	29.02 (3.74)							
AQ	31.56 (5.97)	31.33 (5.37)	19.38 (7.92)	*t*(60) = 6.79	<0.001	*d* = 1.80	*t*(40) = 5.73	<0.001	*d* = 1.75
General cognition									
Full-scale IQ	109.30 (15.20)	112.00 (9.92)	113.57 (11.58)	*t*(60) = −1.42	0.265	*d* = −0.34	*t*(40) = −0.47	0.639	*d* = −0.15
Verbal IQ	112.08 (16.02)	115.05 (11.16)	113.43 (12.35)	*t*(60) = −0.34	0.737	*d* = −0.21	*t*(40) = 0.45	0.658	*d* = 0.14
Performance IQ	104.10 (15.63)	105.38 (12.46)	110.81 (12.38)	*t*(60) = −1.71	0.067	*d* = −0.49	*t*(40) = −1.42	0.121	*d* = −0.44
Social cognition									
Eyes Test (%)	59.69 (8.59)	60.58 (6.19)	67.72 (7.73)	*t*(60) = −3.60	0.001	*d* = −0.98	*t*(40) = −3.31	0.002	*d* = −1.03
FER (%)	71.49 (7.89)	69.54 (8.35)	73.21 (7.17)	*t*(60) = −0.84	0.406	*d* = −0.23	*t*(40) = −1.53	0.134	*d* = −0.47
FER-BP (%)	45.53 (10.39)	41.77 (11.13)	50.60 (6.56)	*t*(57.2) = −2.03	0.023	*d* = −0.60	*t*(32.4) = −3.13	0.004	*d* = −1.00
SR (%)	83.41 (17.12)	80.95 (19.98)	90.48 (8.05)	*t*(59.7) = −2.21	0.174	*d* = −0.56	*t*(26.3) = −2.03	0.156	*d* = −0.68
Detail-focused processing									
EFT (s)	102.46 (11.48)	103.70 (10.73)	105.08 (6.28)	*Z* = −0.19	0.888	*r* = −0.03	*Z* = 0.29	0.697	*r* = 0.05
BD (%)	90.27 (15.27)	90.64 (14.89)	78.38 (15.41)	*t*(60) = 2.67	0.005	*d* = 0.78	*t*(40) = 2.62	0.012	*d* = 0.81
Executive function									
WCST (%)	78.89 (14.38)	78.75 (14.95)	87.64 (2.83)	*Z* = 3.37	0.001	*r* = 0.52	*Z* = 2.67	0.008	*r* = 0.41
Tower test (raw score)	19.95 (5.24)	21.71 (5.15)	21.43 (5.35)	*t*(60) = −1.04	0.307	*d* = −0.28	*t*(40) = 0.18	0.861	*d* = 0.05
CPT (T-score)	55.17 (8.41)	56.90 (7.92)	57.33 (7.81)	*Z* = −1.42	0.157	*r* = −0.18	*Z* = −0.45	0.650	*r* = −0.07
Working memory									
LNS (scaled score)	10.20 (3.68)	10.71 (2.70)	11.67 (2.78)	*Z* = 1.32	0.187	*r* = 0.17	*Z* = 0.65	0.515	*r* = 0.10
VS (%)	71.39 (13.95)	73.63 (9.68)	79.49 (8.46)	*Z* = 2.37	0.018	*r* = 0.30	*Z* = 2.07	0.038	*r* = 0.32
Long-term memory									
LM (%)	65.55 (15.51)	67.24 (11.43)	63.43 (12.60)	*t*(60) = 0.52	0.606	*d* = 0.15	*t*(40) = 1.03	0.311	*d* = 0.32
RCFT (%)	68.73 (13.63)	69.05 (16.86)	79.30 (11.61)	*Z* = 2.75	0.006	*r* = 0.35	*Z* = 1.94	0.052	*r* = 0.30
PM (%)	83.06 (13.94)	87.30 (8.81)	92.06 (6.23)	*Z* = 2.55	0.011	*r* = 0.32	*Z* = 1.81	0.071	*r* = 0.28
Verbal ability									
VFT (words)	84.61 (21.92)	87.38 (17.57)	95.05 (20.05)	*t*(60) = −1.83	0.073	*d* = −0.50	*t*(40) = −1.32	0.195	*d* = −0.41
Letter fluency	37.20 (11.72)	37.71 (9.63)	42.86 (10.90)	*t*(60) = −1.84	0.131	*d* = −0.50	*t*(40) = −1.62	0.236	*d* = −0.50
Category fluency	47.41 (12.54)	49.67 (11.24)	52.19 (10.55)	*t*(60) = −1.45	0.243	*d* = −0.41	*t*(40) = −0.75	0.791	*d* = −0.23
Processing speed									
DS (scaled score)	10.78 (4.26)	11.38 (3.91)	12.81 (2.91)	*t*(60) = −1.42	0.055	*d* = −0.57	*t*(40) = −1.34	0.186	*d* = −0.42

*ASD* autism spectrum disorder group, *ASD-ND* autism spectrum disorder participants not taking any psychotropic medication (non-drug), *CON* control group, *SD* standard deviation, *ES* effect size, *CARS* Childhood Autism Rating Scale, *CARS2-HF* CARS, second edition, high-functioning version, *AQ* Autism-Spectrum Quotient, *IQ* intelligence quotient, *Eyes Test* “Reading the Mind in the Eyes” test, revised version, *FER* Facial Emotion Recognition task, *FER-BP* Facial Emotion Recognition from briefly presented expressions, *SR* Self-Reference task, *EFT* Embedded Figures Test, *BD* Un/segmented Block Design task, *WCST* Wisconsin Card-Sorting Test, *CPT* Continuous Performance Test, *LNS* Letter-Number Sequencing task, *VS* Visuospatial Span task, *LM* Logical Memory task, *RCFT* Rey Complex Figure Test, *PM* Prospective Memory task, *VFT* Verbal Fluency task, *DS* Digit Symbol task

The control (CON) group consisted of 21 typically developing adults who were matched with 21 ASD participants not taking any psychotropic medication (non-drug; ASD-ND group) for age, gender, years of education, and full-scale, verbal, and performance IQs (all *p* ≥ .12). The ASD and CON groups did not differ in terms of these variables (all *p* ≥ .06). The IQs of all participants were measured using the Japanese version of the Wechsler Adult Intelligence Scale, third edition (WAIS-III: Fujita et al. 2006; Wechsler 1997). Additionally, all participants completed the Japanese version of the Autism-Spectrum Quotient (AQ) questionnaire (Baron-Cohen et al. 2001b; Wakabayashi et al. 2004), a 50-item self-rated scale measuring autistic traits. The AQ scores of participants in the ASD and ASD-ND groups were significantly higher than those in CON participants (all *p* < .001) as expected. The demographic characteristics, IQs and AQ of participants are also provided in Table 1.

Exclusion criteria for all participants included a history of or a current psychotic disorder, substance or alcohol abuse, traumatic head injury, a genetic disorder associated with autism (e.g., fragile X syndrome, tuberous sclerosis), intellectual disability, or any other medical condition significantly affecting brain function (e.g., epilepsy).

All procedures in this study were approved by the Ethics Committee of the Graduate School and Faculty of Medicine at Kyoto University and were performed in accordance with the ethical standards in the 1964 Declaration of Helsinki and its later amendments. All participants provided written informed consent to participate in the study.

### Measures

According to evidence of atypicality in individuals with ASD, we selected measures in the following cognitive domains of neuro- and social cognition. The social cognitive domains were mentalizing, social perception, and self-referential cognition (Lai et al. 2014). The neurocognitive domains were detail-focused processing (Happé and Frith 2006), executive function (Hill 2004; Kenworthy et al. 2008), working memory (Williams et al. 2005), long-term memory (Williams et al. 2014; Minshew and Goldstein 2001; Toichi and Kamio 2002, 2003), verbal ability (Rumsey and Hamburger 1988), and processing speed (Nakahachi et al. 2006). Because approximately 4 h was required to complete all cognitive measures, they were divided into two parts and implemented over two continuous or discontinuous days within 15 days in the same sequence, considering that participant fatigue could influence task performance. All cognitive tasks and neuropsychological tests were administered individually by a clinical psychologist trained in standardized testing procedures.

#### Social Cognitive Measures

##### Mentalizing

We used the Eyes Test (Baron-Cohen et al. 2001a) to measure mentalizing ability. The Eyes Test, comprising 36 items, required participants to infer mental state from information in the eye region and to select the most suitable adjective from four choices. The measure used in this study was percentage of items answered correctly (accuracy).

##### Social perception

The FER task, with which atypicality in ASD is commonly reported (see Harms et al. 2010 for a review), was used to assess ability to perceive emotions. This task used a label-matching paradigm that was previously used by Sato et al. (2002) and Uono et al. (2011, 2013). We used pictures of 48 young adults with facial expressions depicting six basic emotions (anger, disgust, fear, happiness, sadness, and surprise) from a standard photograph set (Matsumoto and Ekman 1988). Participants were shown each picture presented on a computer monitor in a predetermined random order for 2000 ms, which is the duration typically developing adults require to recognize emotions from facial expressions correctly (Wallace et al. 2015), and asked to choose the one that best described the person’s emotion, of the six labels of basic emotions presented next to each picture, within 10 s. No feedback was provided about performance. Participants viewed each emotional expression eight times, resulting in a total of 48 trials for each participant. Half of the trials presented pictures of 24 people with subtle (low-intensity, 60%) facial expressions that have been reported to be sensitive to FER deficits in high-functioning adults with ASD (Doi et al. 2013; Smith et al. 2010). The pictures with subtle facial expressions were rendered by smoothly blending neutral and emotional expressions taken from the same individual at the ratio of four to six, using commercial ‘morphing’ software (FantaMorph 5, Abrosoft). In the other half of the trials, we presented original pictures of other people (high-intensity, 100%). In each intensity condition, half of the pictures were of females and half of males; also, half were of Japanese and half were of Caucasians. Prior to testing, we established that all participants understood the meaning of the emotional labels and the task instructions, and participants engaged in two training trials to become familiar with the procedure. Accuracy was used as the measure.

Before this FER testing, participants performed a task to recognize emotions from briefly presented facial expressions (FER-BP; Clark et al. 2008). The FER-BP task has been used to measure the ability to extract emotional information rapidly, which relies more on automatic processing and less on the use of verbal or other top-down strategies that could compensate for inefficient performance on emotional perception in high-functioning individuals with ASD (see Harms et al. 2010). This task was identical to the FER task except for the very short duration of picture presentation, 50 ms, which is about the same duration used for micro expressions (Ekman 2004). The pictures, which were the same as those in the FER, were presented in a different order from in the FER.

Following the two FER tasks, the face perception task was performed. In this task, participants were shown pictures of 12 people with neutral expressions for 50 ms in a predetermined random order, and asked to choose one that had been presented just before, of two pictures with neutral expressions, within 10 s. The 24 people were selected from the 48 whose pictures were used in the FER tasks, keeping the same ratios of females to males and Caucasians to Japanese. All participants in both groups, with the exception of one ASD participant who made only one incorrect answer, got all the answers right. Thus, the accuracy was 99.8% in the ASD group, indicating that ASD participants could extract sufficient information about major facial features to recognize individuals from pictures presented for such a short duration.

##### Self-referential cognition

We used the Self-Reference task (Toichi et al. 2002; Yoshimura and Toichi 2014) to assess the processing of self-referential information. This memory task consists of the learning phase, during which participants encode 30 words (targets) on three levels of processing, and an incidental test phase. Participants were initially presented with each yes-or-no question on a computer monitor for 8 s. These questions addressed each subsequently presented target word (an adjective describing a personality trait), which was presented for 2 s. Participants were then required to answer within 5 s. Three types of questions that made participants to encode each target on the different levels were as follows: phonological (“Does the word rhyme with xxx?”), semantic (“Is the meaning of the word similar to xxx?”), and self-referential (“Does the word describe you?”). Ten questions were arranged for each of three types. In the incidental recognition test, which immediately followed the learning-phase, participants were required to identify the 30 targets from a list of 90 words, including 60 distractors, within 5 min. The percentage of correctly recognized words in self-referenced target words was the measure used.

#### Neurocognitive Measures

##### Detail-focused processing

The Embedded Figures Test (Witkin 1950) was used to measure attention to detail in visuospatial cognition. This task, comprising 24 items, required participants to locate a geometrically simple shape (target) within a larger complex design. A time limit of 120 s was set in accordance with Lai et al. (2012), and the mean of the remaining time after finding each target was used as the measure (in s; 0–120).

We also used the Un/segmented Block Design (BD) task (Shah and Frith 1993) to assess superiority in detail-focused processing style. In the BD task, participants were asked to replicate designs using four blocks as quickly as possible, as with the BD subtest of the WAIS-III. First, participants were presented with eight unsegmented (whole) designs in a fixed order and, in the second half, segmented (separate) sets of the same designs were presented in the same order. The percentage of response time to construct all segmented designs, with time to construct unsegmented designs as a baseline for comparison, was the measure used.

##### Executive function

The Wisconsin Card Sorting Test (Heaton et al. 1993) was used as a measure of cognitive flexibility. This task requires participants to match response cards, a maximum of 128 cards, to the four stimulus cards in one of three categories (color, form, or number) on the basis of only the examiner’s feedback as to whether each response was right or wrong. The category changed without warning when ten consecutive cards were sorted correctly, until six categories were completed. The measure used was the percentage of conceptual-level responses, which were consecutive correct responses occurring in runs of three or more.

The Tower Test of the Delis-Kaplan Executive Function System (Delis et al. 2001) was used to measure planning ability. In the Tower Test, participants were asked to build a tower using variously sized disks, a maximum of five disks, stacked on three pegs in the fewest number of moves possible according to the following rules: (1) move only one disk at a time and (2) never place a big disk on top of a little one. The total achievement score (sum of the raw scores; 0–30) was used as the measure.

We also used the Conners Continuous Performance Test, third edition (Conners 2014), to assess inhibition. This is a Go/No-Go task, in which participants are required to left-click when any letter except the letter “X” (target) appeared on a computer monitor (Go trial), and to give no response to “X” (No-go trial). The measure used was the age-adjusted T-score of detectability, which was reversed, so that higher scores indicated better performance.

##### Working memory

The Letter-Number Sequencing subtest of WAIS-III was used as a measure of auditory working memory. In this task, following the auditory presentation of serial numbers and letters, participants were required to first give the numbers in ascending order and then the letters in alphabetical order. The age-adjusted scaled score was used as the measure.

We used the Visuospatial Span subtest of the Japanese version of the Wechsler Memory Scale, Revised (WMS-R; Sugishita 2001, Wechsler and Stone 1987) to assess visuospatial working memory. In the forward condition, after the examiner had tapped the cubes in a predetermined sequence, participants were asked to repeat the sequence. In the backward condition, the sequence had to be repeated backwards. The proportion of items correctly repeated was used as the measure.

##### Long-term memory

The Logical Memory subtest of the WMS-R was used to measure verbal memory. In this task, participants were asked to recall a story heard as accurately as possible, immediately following auditory presentation of the story (immediate recall) and at least 30 min after the first recall (delayed recall). The study used story A from the WMS-R. The measure used was the proportion of words correctly recalled at the time of delayed recall.

The Rey Complex Figure Test (Meyers and Meyers 1995) was used to assess visuospatial memory. This task requires participants to draw a design relying on memory, 3 min after completing the copy trial (immediate recall) and at least 30 min after the first recall (delayed recall). Each unit of the figure reproduced was scored according to the criteria of Meyers and Meyers (1995), which were originally developed by Rey (1941). The proportion of the maximum score was used as the measure.

We used the Prospective Memory tasks of the Memory for Intentions Screening Test (MIST; Raskin et al. 2010). In this task, participants were required to do or say certain things at assigned times (e.g. “In 15 min, tell me that it is time to take a break”) during a word search task that lasts about 25 min and serves as a distractor to prevent rehearsal. This task includes eight items, counterbalanced for length of delay (2 or 15 min), response type (verbal or action), and cue type (time-based or event-based). The MIST also contains the 24-h delayed task. This time-based task had only one item that required participants to call and tell the tester how many hours they slept last night. Each item was assigned a score from 0 to 2, adding up to a maximum of 18. The proportion of the maximum score was the measure used.

##### Verbal ability

The Verbal Fluency Task (VFT; Ito et al. 2004) was used to measure verbal generativity. In the VFT, participants were asked to generate as many words that began with a given letter or fell into a given category as possible within 60 s in each trial. The Japanese syllables “a,” “ka,” and “shi” and the categories of “animal,” “sport,” and “occupation” were used in six separate trials. The total number of words generated was scored.

##### Processing speed

The Digit Symbol subtest of the WAIS-III was used as a measure of processing speed. This task requires participants to copy symbols paired with digits as quickly as possible in the empty boxes below a random sequence of digits within 120 s. The measure used was the age-adjusted scaled score.

#### Functional Outcome Measures

##### Adaptive functioning

The Japanese version of the Vineland Adaptive Behavior Scales, second edition (Vineland-II; Sparrow et al. 2005; Tsujii et al. 2014), was used to assess adaptive functioning in ASD participants. Vineland-II provides standard scores, which have a mean of 100 and a standard deviation of 15, in an overall adaptive behavior composite and subdomains including Communication, Daily Living Skills, and Socialization in adults. The scores in 30 participants whose parents or spouses cooperated and gave written informed consent were available. Vineland-II was administered on or within 2 months after the first day of the cognitive testing.

##### Social functioning

Overall social functioning in all participants was rated on an ascending scale of zero to four based on four components: residential status, employment/education, intimate relationship, and friendships, according to previous studies (Farley et al. 2009; Howlin et al. 2004, 2013). Information needed for the rating was obtained through a structured interview with participants on the first day of the cognitive testing. Based on criteria in previous studies (Farley et al. 2009; Howlin et al. 2013; Taylor and Selzer 2012) and the proportion of ASD participants meeting those, we established the following criteria for a ‘better’ outcome, to avoid a subjective judgment made by participants and raters, on each component: s/he lives by her/himself, or with his/her spouse and/or children (residential status), s/he is employed full-time or part-time more than 10 h/week, or in a graduate or postsecondary education program (employment/education), s/he is married or has continued an intimate relationship for more than 1 year (intimate relationship), and s/he has met one or more friends in the past 3 months (friendships). A composite rating of social outcome was scored by counting the number of items fulfilled by each participant.

### Statistical Analysis

Data were analyzed in three steps. Statistical analyses were conducted using the SPSS software (ver. 22). All analyses were two-tailed, and α was set at 0.05.

Step 1: All variables of cognitive measures were tested for a normal distribution with the Shapiro–Wilk test. Then, for measures that were not normally distributed, non-parametric Mann–Whitney *U* tests were used to investigate between-group differences. Where appropriate, we used independent *t* tests for group comparisons. Additionally, paired *t* tests were used to compare the scores in the three subdomains of Vineland-II.

Step 2: Pearson’s correlations were calculated to assess associations between measures of cognition and adaptive functioning, and between measures of neuro- and social cognition.

Step 3: To see whether measures of adaptive functioning could predict social functioning in ASD participants, we performed a step-wise multiple linear regression analysis including Vineland-II composite score, age, gender, years of education, medication, AQ, and CARS2-HF score as independent variables (predictors), and overall social functioning score as the dependent variable (outcome). Then, to identify specific cognitive abilities predicting adaptive functioning, step-wise multiple linear regression analyses were conducted between all measures on neuro- or social cognition as independent variables and Vineland-II composite as the dependent variable. If the predictive relationships between measures of both neuro- and social cognition and adaptive functioning were significant, we conducted regression analyses, which involved one measure each of neuro- and social cognition, and a bootstrapping method to test the mediation path (indirect effect of independent variable on dependent variable through a mediator), using the SPSS PROCESS macro (Hayes 2013). An estimate of the indirect effect was the mean computed using 5000 bootstrap samples, and the 95% bias-corrected confidence interval was constructed from the sampling distribution. If zero was within the 95% confidence interval, the mediation effect was considered to be significant at *p* < .05, rejecting the null hypothesis that the mediation effect was zero (Preacher and Hayes 2004, 2008).

## Results

### Group Comparison on Cognition

Table 1 presents the results of *t* tests and Mann–Whitney *U* tests. Regarding social cognition, performances on the Eyes Test (*p* < .01) and FER-BP (*p* < .01) were impaired in ASD-ND participants versus CON participants. Those performances in the ASD group were also impaired in comparison with CON (*p* < .01, *p* < .05, respectively). However, performance in the CON and ASD groups did not differ on the other measures in social cognition (all *p* ≥ .13).

Regarding neurocognition, performances on BD (*p* < .05), the Wisconsin Card Sorting Test (*p* < .01), and the Visuospatial Span task (*p* < .05) were impaired in ASD-ND versus CON participants. In addition to those measures (*p* < .01, *p* < .01, *p* < .05, respectively), performances on the Rey Complex Figure Test (*p* < .01) and the Prospective Memory task (*p* < .05) in the ASD group were also impaired in comparison with those in the CON group. However, performance in terms of these two measures in ASD-ND participants did not differ from that in CON participants (all *p* ≥ .05). Performance in the CON and ASD groups did not differ on the other measures in neurocognition (all *p* ≥ .05).

### Functional Outcomes

Functional outcome characteristics in ASD participants are presented in Table 2. Regarding adaptive functioning, Vineland-II scores were obtained for 30 participants with ASD. The mean composite score was 71.33 (range = 20–109). The mean subdomain scores were 74.70 (range = 31–103) for communication, 84.60 (range = 31–110) for daily living skills, and 71.20 (range = 38–101) for socialization. Paired *t* tests comparing subdomain scores demonstrated that the daily living skills were significantly higher than communication (*t*(29) = 4.38, *p* < .001, *d* = 0.49) or socialization (*t*(29) = 4.65, *p* < .001, *d* = 0.64). The domain scores on communication and socialization did not differ (*t*(29) = 1.53, *p* = .14, *d* = 0.16).

**Table 2:** Functional outcome in ASD participants

Characteristic	Mean (SD)/N (%)
Age categories	
18–24	20 (48.8)
25–34	13 (31.7)
35–44	7 (17.1)
45–54	1 (2.4)
IQ categories	
70–89	4 (9.8)
90–109	16 (39.0)
110–129	17 (41.5)
≥130	4 (9.8)
Vineland-II (*n* = 30)	
Composite score	71.33 (24.84)
Communication	74.70 (20.30)
Daily living skills	84.60 (19.85)
Socialization	71.20 (22.25)
Residential status	
Living by oneself	13 (31.7)
Living with spouse	2 (4.9)
Living with spouse and child(ren)	4 (9.8)
Living in parents’ home	22 (53.7)
Employment (*n* = 28; excluding students)	
Employed full-time	10 (35.7)
Employed part-time ≥10 h/week	2 (7.1)
Employed <10 h/week	2 (7.1)
Supported/sheltered employment	4 (14.2)
No vocational activity	10 (35.7)
Education	
Graduate student	2 (4.9)
Postsecondary educational program	11 (26.8)
College graduate	21 (51.2)
Junior college graduate	4 (9.8)
High school graduate	2 (4.9)
College dropout	3 (7.3)
Relationship (current)	
Married	6 (14.6)
Long-time intimate relationship ≥1 year	2 (4.9)
Intimate relationship <1 year	2 (4.9)
No intimate relationship	31 (75.6)
Friendships	
One or more friends	29 (70.7)
No specific friendship	12 (29.3)
Overall social outcome	
Very good (4)	4 (9.8)
Good (3)	12 (29.3)
Fair (2)	11 (26.8)
Poor (1)	7 (17.1)
Very poor (0)	7 (17.1)

*n* = 41, *SD* standard deviation; Vinland-II scores were obtained for 30 ASD participants; the item of employment gives information about 28 ASD participants, excluding students. *ASD* autism spectrum disorder, *IQ* intelligence quotient, *Vineland-II* Vineland adaptive behavior scale, second edition

For social functioning, based on residential status, employment/education, intimate relationship, and friendships (Farley et al. 2009; Howlin et al. 2004, 2013), we rated the overall social outcome in each participant using a five-point scale. The outcomes in 4 (9.8%) ASD participants were classified as “very good” (4 points), 12 (29.3%) were “good” (3 points), 11 (26.8%) were “fair” (2 points), 7 (17.1%) were “poor” (1 point), and 7 (17.1%) were “very poor” (0 points). The median score for social outcome in the ASD group was 2 points (fair). In the CON group, 8 (38.1%) participants were classified as “very good,” 13 (61.9%) as “good,” and no participant was rated fair, poor, or very poor, resulting in a median of 3 points (good).

### Associations Between Cognitive and Adaptive Functioning

Vineland-II composite scores showed the strongest correlation with performance on VFT (*p* < .01) and were correlated significantly with full-scale (*p* < .05) and verbal IQs (*p* < .05), as well as with performances on FER (*p* < .01), the Prospective Memory task (*p* < .05), and the Digit Symbol task (*p* < .05). No other correlation was statistically significant (all *p* ≥ .05).

### Associations Between Neuro- and Social Cognition

Correlations between measures of neuro- and social cognition in ASD participants are presented in Table 3. Regarding the Eyes test, the correlations with full-scale (*p* < .05) and verbal IQs (*p* < .05), and the Prospective Memory task (*p* < .05) were significant. However, FER, FER-BP, and the Self-Reference task showed no correlation with full-scale or verbal IQs (all *p* ≥ .06). For FER, the correlation with VFT was significant (*p* < .05). The correlations between FER-BP and BD (*p* < .05) and between the Self-Reference task and the Continuous Performance Test (*p* < .01) were also significant. No other correlation was significant (all *p* ≥ .06).

**Table 3:** Correlations between neuro- and social cognition in ASD participants

	Eyes test	FER	FER-BP	Self-reference
General cognition				
Full-scale IQ	0.35*	0.21	0.29	−0.10
Verbal IQ	0.32*	0.20	0.30	0.04
Performance IQ	0.25	0.16	0.21	−0.25
Detail-focused processing				
Embedded figures test	0.14	0.07	0.11	0.06
Un/segmented block design	0.16	0.30	0.34*	−0.25
Executive function				
Wisconsin card sorting test	0.24	−0.04	−0.14	−0.16
Tower test	0.08	−0.09	−0.13	−0.14
Continuous performance test	0.19	0.03	−0.14	0.47**
Working memory				
Letter-number sequencing	0.14	0.27	0.00	−0.14
Visuospatial span	0.18	0.15	0.07	−0.06
Long-term memory				
Logical memory	0.09	0.17	0.19	0.03
Rey complex figure test	−0.03	−0.10	0.04	0.09
Prospective memory	0.40*	0.13	0.06	0.02
Verbal ability				
Verbal fluency	0.28	0.31*	−0.05	0.03
Processing speed				
Digit symbol	0.14	0.25	−0.08	−0.26

*Correlation was significant at .01 < *p* < .05 level, **correlation was significant at *p* < .01 level

*n* = 41. *Eyes Test* “Reading the Mind in the Eyes” test, revised version, *FER* Facial Emotion Recognition task, *FER-BP* Facial Emotion Recognition from briefly presented expressions, *IQ* intelligence quotient

### Regression of Functional Outcome

Table 4 presents the results of a step-wise multiple linear regression analysis, which included social functioning score as a dependent variable, and Vineland-II composite score, age, gender, years of education, medication, AQ, and CARS-HF score as independent variables. In the final model, Vineland-II composite score was the only significant predictor (*p* < .001), which accounted for 64% of the variance in social functioning (*p* < .001). No other variable was significant in combination with Vineland-II.

**Table 4:** Step-wise multiple linear regression analysis, including social functioning score as the dependent variable, Vineland-II composite score, age, gender, years of education, medication, AQ, and CARS2-HF as independent variables

	*β*	Statistic	*p* value	95% CI	*R* ^2^
Model		*F*(1, 28) = 48.88	<0.001		0.64
Vineland-II composite score	0.04	*t* (28) = 7.00	<0.001	0.03–0.06	

*n* = 30. *Vineland-II* Vineland adaptive behavior scale, second edition, *AQ* autism spectrum quotient, *CARS2-HF* childhood autism rating scale, second edition, high-functioning version, *CI* represents confidence interval, *R* ^2^ represents variance explained by the independent variable in the model

Table 5 presents step-wise multiple linear regression analyses that included the Vineland-II composite score as a dependent variable and all measures of neuro- or social cognition as independent variables. Regarding social cognition, FER was the only significant predictor (*p* < .01), accounting for 35% of the variance in adaptive functioning (*p* < .01). For neurocognition, the final model demonstrated that performances on both VFT (*p* < .001) and BD (*p* < .05) were significant predictors of the Vineland-II score, accounting for 47% of the variance in adaptive functioning (*p* < .001). No other cognitive measure was significant in combination with FER, or VFT and BD.

**Table 5:** Step-wise multiple linear regression analysis, including Vineland-II composite score as the dependent variable and measures of social cognition or neurocognition as independent variables, and testing for mediation of social cognition in the relationship between neurocognition and adaptive functioning

	*β*	Statistic	*p* value	95% CI	*R* ^2^
Model: Vineland-II on social cognition		*F*(1, 28) = 15.29	0.001		0.35
Facial emotion recognition	1.90	*t* (28) = 4.36	0.001	0.91–2.90	
Model: Vineland-II on neurocognition		*F*(2, 27) = 11.93	<0.001		0.47
Verbal fluency	0.64	*t* (27) = 4.36	<0.001	0.34–0.95	
Un/segmented block design	0.47	*t* (27) = 2.18	0.039	0.03–0.91	
Model: Vineland-II on VFT and FER		*F*(2, 27) = 12.77	<0.001		0.49
Verbal fluency	0.44	*t*(27) = 2.64	0.014	0.10–0.79	
Facial emotion recognition	1.23	*t*(27) = 2.40	0.023	0.18–2.28	
Indirect effect of VFT through FER	0.20			0.03–0.49	
Model: Vineland-II on BD and FER		*F*(2, 27) = 9.19	<0.001		0.41
Un/segmented block design	0.35	*t*(27) = 1.54	0.136	−0.12 to 0.83	
Facial emotion recognition	1.80	*t*(27) = 3.75	0.001	0.81–2.78	
Indirect effect of BD through FER	0.12			−0.20 to 0.53	

*n* = 30. *Vineland-II* Vineland adaptive behavior scale, second edition, *FER* Facial Emotion Recognition task, *VFT* Verbal Fluency Task, *BD* Un/segmented Block Design task, *CI* represents confidence interval, *R* ^2^ represents variance explained by the independent variable in the model

### Analyses of Mediation

**Fig. 1:**
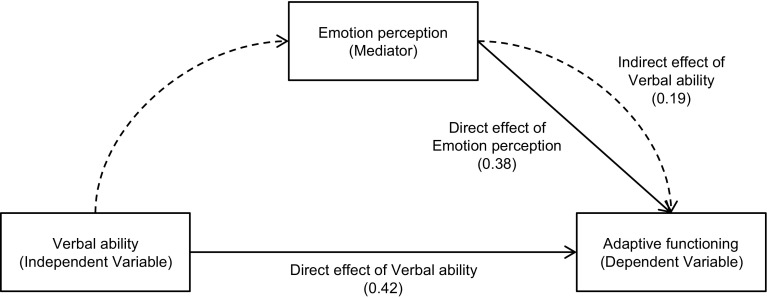
Illustration of the mediation model including adaptive functioning as the dependent variable, verbal ability as the independent variable, and emotion perception as the mediator. *Arrows* indicate the direction of prediction. *Numbers* on *arrows* indicate standardized regression weights. *Continuous arrows* represent the direct effects. *Dotted arrows* represent the indirect effect of verbal ability, which is part of the direct effect of emotion perception on adaptive functioning

Because we could identify significant predictors of adaptive functioning in neuro- (verbal ability and detail-focused processing) and social cognition (emotion perception), regression analyses were conducted with the bootstrapping method to examine the validity of the mediation models involving FER and VFT or BD. The results of regressions are presented in Table 5. The combination of VFT and FER accounted for 49% of the variance in adaptive functioning (*p* < .001), and the bootstrapping method showed that zero was not within the 95% CI of the indirect effect of VFT on Vineland-II through FER, demonstrating a significant effect of mediation (*p* < .05; Fig. 1). The combination of BD and FER accounted for 41% of the variance in adaptive functioning (*p* < .001), but the bootstrapping method demonstrated that zero was within the 95% CI of the indirect effect of BD on Vineland-II through FER, indicating that the mediation effect was not significant (*p* ≥ .05).

## Discussion

This cross-sectional study tested three hypotheses concerning predictive relationships among neuro- and social cognition and adaptive and social functioning in adults with ASD and average or higher IQ. Our results support all three hypotheses that (1) adaptive functioning is an outcome indicator related very closely to social functioning, (2) the combination of verbal ability in neurocognition and emotion perception in social cognition accounts for substantial variance in adaptive functioning, and (3) emotion perception partially mediates the predictive relationship between verbal ability and functional outcome. To our knowledge, these exploratory findings represent the first reported evidence of a triadic relationship among neuro- and social cognition and adaptive functioning in high-functioning adults with ASD. In what follows, we begin by establishing the generalizability of our results in this population.

### Characteristics of Cognition in ASD Participants

ASD participants in the current study showed atypicalities in several abilities in social cognition, including mentalizing and emotion perception, and in neurocognition, including detail-focused processing, cognitive flexibility, and visuospatial working memory. Atypical or inefficient performance on other measures was not seen in this ASD sample, which does not contradict the accumulated knowledge on this issue. Given the heterogeneity in ASD, large variations in cognitive performance between and within studies of this condition should be expected.

Regarding social cognition, although many studies have shown deficits on FER in individuals with ASD, some high-functioning adults with ASD can recognize prototypical facial expressions as well as typically developing adults, presumably capitalizing on their cognitive resources (see Harms et al. 2010). The results that ASD participants could perform well on FER, but poorly on FER-BP, support the notion that effortful processing can compensate for inefficiency in emotion perception in high-functioning adults with ASD. The correlation between performances on VFT and FER, but not FER-BP, only in ASD participants (correlation in CON: *r* = −0.12, *p* > .10) appears to indicate that verbal ability may contribute to the effort after initial processing in facial emotion perception. Additionally, the correlation between BD and FER-BP, only in ASD participants (correlation in CON: *r* = 0.09, *p* > .10), suggests that local or feature-based processing is an advantage for ASD individuals in extracting emotional information from facial expressions rapidly. Performances on FER and on the Self-Reference task in this high-functioning ASD sample were generally comparable to those in previous studies (Uono et al. 2013; Yoshimura and Tocihi 2014).

Regarding neurocognition, similarly, performances in planning (Losh et al. 2009), inhibition (Schmitz et al. 2006), verbal working memory (Williams et al. 2005), verbal memory (Ambery et al. 2006), visuospatial memory (Minshew and Goldstein 2001), verbal generativity (Wilson et al. 2014), and processing speed (Lehnhardt et al. 2016) were comparable those in high-functioning adults with ASD reported in previous studies. Prospective memory performance on the MIST (Raskin et al. 2010) in high-functioning adults with ASD has never been investigated. Our results showed that prospective memory performance had a positive correlation with verbal IQ (*r* = 0.57, *p* < .001) only in ASD participants. Performances on the Embedded Figures Test (*r* = 0.43, *p* < .01), the Tower Test (*r* = 0.43, *p* < .01), the Continuous Performance Test (*r* = 0.32, *p* < .05), the Letter-Number Sequencing task (*r* = 0.53, *p* < .001), and the Logical Memory task (*r* = 0.57, *p* < .001) also correlated positively with verbal IQ only in the ASD group (correlation in CON: all |*r*| ≤ 0.33, all *p* ≥ .14), suggesting that atypical performance on those tasks in individuals with superior verbal intelligence might be inconspicuous or be compensated for. In the case of VFT, performance on the task correlated positively with verbal IQ in ASD participants (*r* = 0.50, *p* < .01) and those in the CON group (*r* = 0.57, *p* < .01), indicating the validity of considering the VFT score as a variable representing verbal ability.

### Characteristics of Outcomes in ASD Participants

The fact that overall social functioning in more than 60% of the adults in this ASD sample was poorer than in CON participants demonstrates the difficulty in adjusting to the community for high-functioning adults with ASD. The distributions of the composite scores in ASD participants generally corresponded to those in adults with verbal IQ >70 (good or very good 42.9%, fair 28.6%, poor or very poor 28.6%), as reported by Howlin et al. (2004). The proportions of individuals living independently, involved in regular full-time paid work, and married or continuing an intimate relationship were comparable with those in a Canadian sample (31.3, 42.9, and 25.0%, respectively; Szatmari et al. 1989) of high-functioning adults with ASD.

The composite scores of adaptive functioning, assessed by Vineland-II (Sparrow et al. 2005), in this ASD sample were also comparable with those in high-functioning adults with ASD in previous studies (Duncan and Bishop 2015; Farley et al. 2009). The profile of adaptive functioning was in accordance with the “autism profile” established previously (e.g., Carter et al. 1998), characterized by lowest scores in socialization, second lowest in communication, and relatively high scores in daily living skills.

The characteristics of cognition and functional outcomes in high-functioning adults with ASD reported in many previous studies were generally replicated in this sample. Thus, we consider that our results are generalizable in this population.

### Predictive Relationship Among Neuro- and Social Cognition and Functional Outcome

As expected, specific abilities in neuro- and social cognition were identified as significant predictors of functional outcome in high-functioning adults with ASD. Along with verbal ability and emotion perception, which were reported to relate to adaptive functioning in previous studies (Liss et al. 2001; Wallace et al. 2011), detail-focused processing style was found from a broad range of neurocognitive domains in this study. Additionally, the large variance in social functioning explained by the composite score of Vineland-II supported the methodological validity of using this measure as an indicator of functional outcome.

Regarding neurocognition, the substantial power of specific abilities in verbal functions in predicting functional outcomes, which had been found in high-functioning children with ASD (Liss et al. 2001), was replicated in this adult sample. Liss et al. (2001) demonstrated that IQs did not contribute to the prediction of adaptive functioning in high-functioning children with ASD when involved in regression analyses along with verbal abilities. Supplementary analyses (simple linear regressions) of data from this adult sample also showed that general cognitive ability accounted for only a moderate proportion of the variance in adaptive functioning (full-scale IQ: *F*(1, 28) = 4.71, *R* ^2^ = 0.14, *p* < .05; verbal IQ: *F*(1, 28) = 4.95, *R* ^2^ = 0.15, *p* < .05; performance IQ: *F*(1, 28) = 2.16, *R* ^2^ = 0.07, *p* > .10). Although it is easy to imagine that social adaptation is disrupted by language disability, even adaptive functioning in adults with normal or higher verbal intelligence appears to be affected by subtler linguistic problems, including reduced verbal generativity, and possibly stereotyped and repetitive use of language, speech idiosyncrasies, and pragmatic deficits. For individuals with ASD who also have deficits in non-verbal expression, such as facial mimicry (Yoshimura et al. 2015), many verbal expressions may be advantageous for building cooperative or friendly relationships. Regarding detail-focused processing, the minor power of prediction suggests that this cognitive style does not independently influence adaptive functioning but supplements the contribution of verbal abilities. This finding appears to support the notion that a detail-focused style in this population is due not so much to a deficit in global processing but superiority in local processing (Happé and Frith 2006). The talent that makes them focus on local features is possibly advantageous for individuals with higher verbal intelligence in their performance of the daily or specialized tasks requiring sensitivity to details of information.

Regarding social cognition, the close relationship between emotion perception and functional outcome in adults with ASD, which was previously reported as a significant correlation (Wallace et al. 2011), was confirmed by regression analyses. Emotion perception is thought to be bound to skills in communication and socialization in adaptive functioning. The result that performance on the FER, but not the FER-BP or Eyes test, was found to be a significant predictor of functional outcome in this ASD sample suggests that social cognitive skills to recognize emotions carefully from prototypical facial expressions are also advantageous for developing personal relationships, although they have the underlying abnormality in the ability to infer the mental state of others. Additional analyses demonstrated that the Vineland-II composite score correlated significantly only with accuracy in the recognition of sad facial expressions (*r* = 0.42, *p* < .05) among the six emotions (the correlations for the others: all *r* ≤ 0.36, all *p* ≥ .05), which is in consistent with the previous finding (see Wallace et al. 2011). The cognitive skills for individuals with ASD to perceive others’ sadness accurately may lead to an increase in kindness, which is important in maintaining reciprocal relationships. Both emotion perception and verbal ability are cognitive abilities related closely to interpersonal communication and interaction. Thus, it may be argued that both verbal and non-verbal, or expressive and receptive, communication skills are crucial for individuals with ASD to adapt to social needs.

The key finding from this study is that emotion perception acts as a mediator of the predictive relationship between verbal ability and adaptive functioning, whereas the relationship between emotion perception and detail-focused processing was not underpinned. Supplementary analyses demonstrated that the mediation effect of emotion perception in the relationship between verbal IQ and Vineland-II composite score was also significant (*β* = 0.27, 95% CI 0.04–0.63, *p* < .05). This triadic relationship means that the considerable predictive value of verbal ability on functional outcome is actually an indirect effect, reflecting the predictive power of emotion perception, the performance of which depends partially on verbal ability. These findings support the validity of the suggestion that verbal ability contributes to the atypical, effortful, and less automatic processing of emotion perception in high-functioning adults with ASD. Higher verbal ability seems to make a positive contribution to analytical thought process involving judgements of others’ emotions based on currently available information, accumulated experiences, and linguistic knowledge acquired from books, the Internet, or what someone says. This ability may also help to elicit information related to emotions during conversation. Atypical effort was also found in VFT in adults with ASD and higher verbal intelligence in an fMRI study (Beacher et al. 2012). The current and previous findings suggest that atypical abilities in recruiting cognitive resources or strategies to compensate for inefficient performance in diminished cognitive domains relate to functional outcomes in high-functioning adults with ASD. This mechanism of compensation is considered to stand on atypical, intertwined relationships among specific abilities in neuro- and social cognition in this population.

### Implications for Treatment Interventions

We intended to provide evidence to open the door to treatment interventions that target cognitive abilities in adults with ASD. Our findings raise the hypothesis that improvements in psychosocial intervention, or cognitive training, on social perception and verbal ability may lead to benefits in functional outcomes in high-functioning adults with ASD, and warrant further investigations into the effects of such interventions for this population. Regarding social perception, some interventional studies had reported improvement effects on cognitive measures in the domains targeted (Bölte et al. 2015; Faja et al. 2012; Golan and Baron-Cohen 2006). Investigations into the effects on social and adaptive functioning are a key challenge for future research. Mediation of social perception between verbal ability and functional outcome suggests that individuals with higher verbal intelligence may receive substantial benefit from interventions targeting this domain. Linguistic intelligence in or above the average range seems to be required to understand linguistic instructions in defining each emotion or using cognitive strategies to read facial cues. As is the case with social perception (Turner-Brown et al. 2008), cognitive training programs that have demonstrated improvements in verbal generativity in individuals with schizophrenia (e.g., Sánchez et al. 2014) may be applicable to this population. Considering compensation by the effortful or strategic processing, intervention programs focusing on compensatory strategies (Twamley et al. 2012) are likely promising in this population, in comparison with those depending on repetitive drill practices. For children with ASD, several intervention studies focusing on social perception and verbal ability have been reported (see Wass and Porayska-Pomsta 2014 for a review). Future research is expected to identify specific cognitive abilities in childhood that longitudinally predict adult outcomes.

### Limitations

Our findings should be interpreted considering the following limitations. First, the current study focused on adults with ASD and average or higher IQ. These inclusion criteria for ASD participants limit the generalizability of our findings to this high-functioning population. Second, this study used a cross-sectional design. Thus, our results do not exactly reveal longitudinal predictions. Although we have theorized that neurocognition affects social cognition and functional outcome, our results cannot rule out the possibility that social cognition and functional outcomes affect neurocognition. The replicability of our findings needs to be examined in longitudinal investigations. Third, only a few measures were used in each cognitive domain in this study, because we sought to identify specific abilities relating to functional outcome among a broad range of neuro- and social cognitive domains. When further focusing on the intervention targets in verbal functions and in social perception or emotion processing and expression, multiple measures in both domains are needed. Atypicalities on behaviors closely related to both domains, including perception and production of prosody (see O’Connor 2012 for a review) and emotion processing on memory (Beversdorf et al. 1998; Gaigg and Bowler 2008, 2009), should be focused on. Future investigations clarifying inconspicuous atypicalities or compensatory mechanisms in those domains will be useful in developing effective interventions for this population. Fourth, our statistical analyses included only the composite score within scores in Vineland-II because this study focused primarily on cognitive predictors of adaptive functioning and their relationships. Investigations into more complex relationship among social functioning, subdomains of adaptive functioning, cognitive domains, and specific abilities are expected in the future. Finally, the relatively small sample size may limit both the generalizability of our results and the statistical power, mainly for group comparisons on cognition.

## Conclusion

In the current study, we identified emotion perception, verbal ability, and detail-focused processing from a broad range of domains in neuro- and social cognition as cognitive predictors of adaptive functioning in adults with ASD and average or higher IQ. Furthermore, a direct test of mediation revealed that emotion perception mediated the predictive relationship between verbal ability and adaptive functioning. This finding represents the first reported evidence of a triadic relationship among neuro- and social cognition and functional outcome in individuals with ASD. In this triadic relationship, not only emotion perception but also verbal ability acted as direct predictors of adaptive functioning and their relationship also had a significant effect, accounting for approximately half of the variance in functional outcome. Our findings appear to provide new insight that not only specific cognitive abilities in neuro- and social cognition but also atypical or compensatory relationship among them contribute to social adaptation in this heterogeneous population. The suggestion that psychosocial interventions targeting social perception and verbal ability will possibly provide benefits in functional outcome should encourage further research concerning cognitive training for adults with ASD.
